# Evaluation of incidence and outcomes of transformed nodular lymphocyte predominant Hodgkin Lymphoma in the United States: a population-based cohort study

**DOI:** 10.1038/s41408-026-01505-y

**Published:** 2026-04-23

**Authors:** John L. Vaughn, Malak Munir, Ashley Cobb, Sravani Rimmalapudi, Narendranath Epperla

**Affiliations:** 1 Division of Hematology & Oncology, NYU Grossman Long Island School of Medicine, New York, NY USA; 2 Karsh Division of Gastroenterology and Hepatology, Cedars-Sinai Medical Center, Los Angeles, CA USA; 3 Division of Hematology and Hematologic Malignancies, Huntsman Cancer Institute, University of Utah, Salt Lake City, UT USA

**Keywords:** Health care, Medical research

**To the Editor**,

Nodular lymphocyte-predominant Hodgkin lymphoma (NLPHL) is an indolent B-cell lymphoma with a distinct biology and favorable prognosis [1]. However, a subset of patients experience histologic transformation (HT) to diffuse large B-cell lymphoma (DLBCL), an event generally associated with adverse outcomes in other indolent lymphomas [2]. The incidence and clinical implications of HT in NLPHL are incompletely defined. Reported transformation rates vary considerably across studies depending on cohort size, follow-up duration, and study design [3–9]. While the prognostic relevance of transformed NLPHL (t-NLPHL) has been reported [3, 7], a large-scale comparative analysis of t-NLPHL and de novo DLBCL is lacking. Hence, we conducted a population-based cohort study using data from the Surveillance, Epidemiology, and End Results-17 (SEER-17) database to examine the incidence of HT in NLPHL and evaluate the survival outcomes of t-NLPHL in the contemporary era compared to non-transformed NLPHL and de novo DLBCL diagnosed during the same period. We included patients aged 18–84 years old who were diagnosed with NLPHL between 2010 and 2022. We identified patients with HT by following patients from the diagnosis of NLPHL until their subsequent diagnosis of DLBCL. All patients had biopsy-confirmed HT. We did not consider cases of HT where there was only clinical suspicion of transformed disease without biopsy confirmation. Survival of t-NLPHL was calculated from the date of diagnosis of the aggressive histology.

The study outcomes were relative survival (RS), overall survival (OS), lymphoma-specific survival (LSS), and the cumulative incidence of death from lymphoma (CIF). RS was defined as the ratio of all-cause survival to expected survival in a comparable group of individuals from the general population. OS was defined as the probability of death from any cause following diagnosis of lymphoma. LSS was defined as the probability of survival when lymphoma was considered the only possible cause of death. The lymphoma-specific CIF was used to account for the competing risk of death from other causes. The study covariates were age, sex, race, stage, B symptoms, prior receipt of chemotherapy, and prior receipt of radiation therapy. These covariates were selected based on previous studies of lymphoma patients.

The study was conducted in compliance with the Declaration of Helsinki. Given the nature of the study (population-based study from a public repository), this study was IRB-exempt. We tested for differences between categorical and continuous variables using Pearson’s chi-squared test and the Wilcoxon rank-sum test, respectively. We estimated median follow-up time using the reverse Kaplan-Meier method. We modeled the study outcomes using flexible parametric survival models with 6 knots for the baseline cumulative hazard. We used multivariable modeling to compare the study outcomes between patients with t-NLPHL and de novo DLBCL. This was done by using HT as the key independent variable and adjusting for the study covariates. We modeled HT as a time-dependent variable using restricted cubic splines with 3 knots. We handled missing data using multiple imputation with chained equations. P-values less than 0.05 were considered significant. Analyses were performed using Stata version 19.5 (College Station, TX). There were 1700 patients with NLPHL included in the study (Table S1). The median age at diagnosis was 46 years [interquartile range (IQR), 32–59]. Most patients were male (*n* = 1118, 66%). After a median follow-up of 5.58 years, the cumulative incidence of HT was 2.59% (*n* = 44/1700). Compared to patients with de novo DLBCL diagnosed during the same time period, patients with t-NLPHL were younger (median age 52 vs 66 years), more likely to be male (73% vs 56%), more likely to be Black (25% vs 8%), and more likely to have advanced stage disease at diagnosis (59% vs 54%).

Patients with NLPHL who experienced HT (*n* = 44) had lower survival estimates compared to patients who never transformed (*n* = 1656). The 5-year RS for patients without a history of HT was 97% (95% CI, 96–98) compared to 88% (95% CI, 73–95) for patients who subsequently transformed. The other survival outcomes were also numerically worse for patients with HT. The 5-year OS rates were 93% (95% CI, 92–94) and 86% (95% CI, 72–93). The 5-year LSS rates were 97% (95% CI, 95–97) and 89% (95% CI, 76–95). The 5-year CIF estimates were 3% (95% CI, 3–5) and 11% (95% CI, 5–24). The corresponding survival curves are shown in Fig. 1, showing decreased survival after the first year of diagnosis in patients who transformed.

**Fig. 1:**
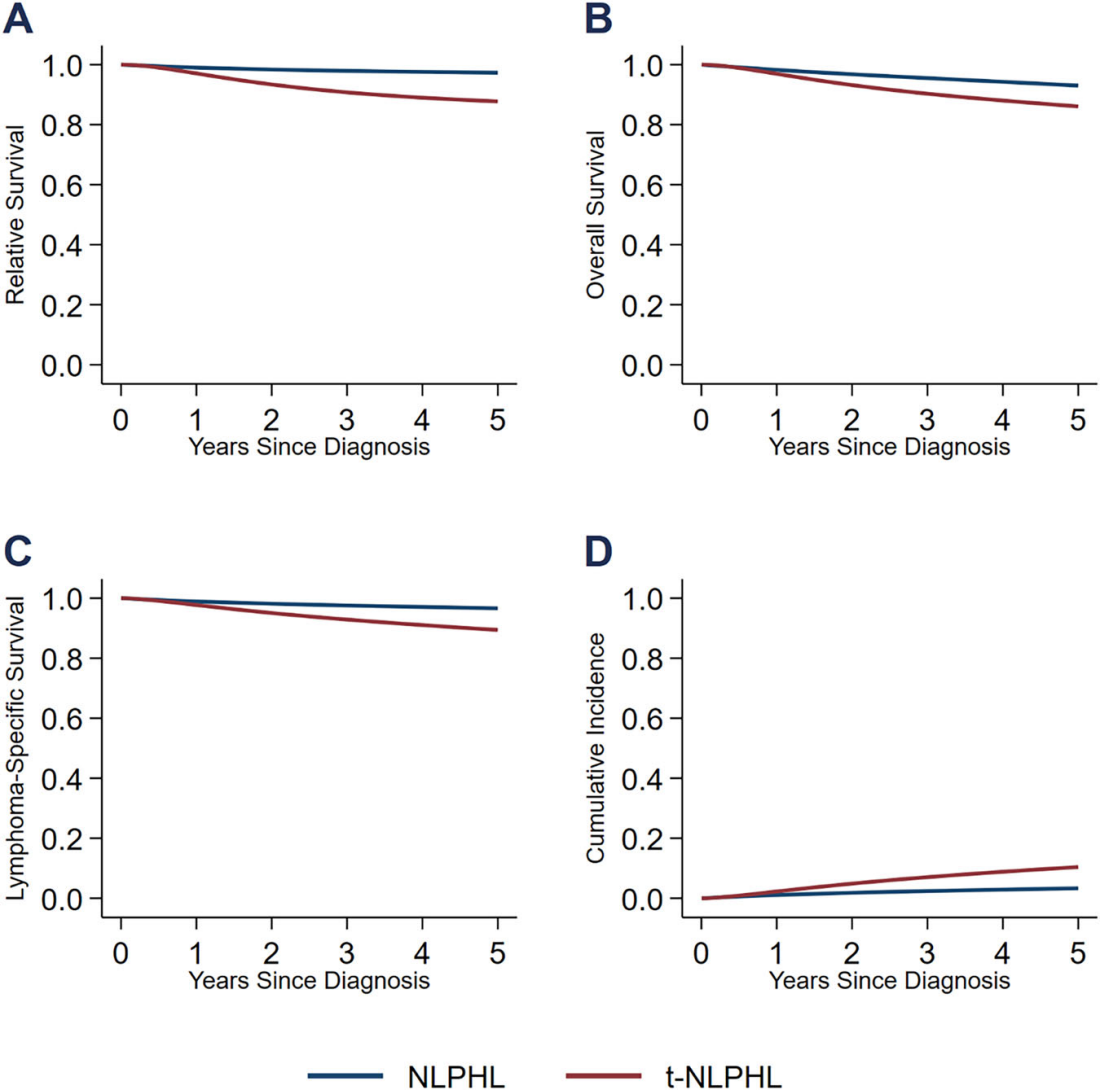
Outcomes of Patients with NLPHL with and without HT. **A** Relative survival. **B** Overall survival. **C** Lymphoma-specific survival. **D** Cumulative incidence of death from lymphoma.

Next, we compared transformed NLPHL (*n* = 44) with de novo DLBCL (*n* = 61,784) diagnosed during the same time period. Patients with t-NLPHL had higher survival rates than patients with de novo DLBCL. The 5-year RS rate for patients with de novo DLBCL was 67% (95% CI, 67–68%) compared to 84% (95% CI, 68–92%) for patients with t-NLPHL. The 5-year OS rates were 58% (95% CI, 58–58%) and 81% (95% CI, 66–90%). The 5-year LSS rates were 68% (95% CI, 67–68) and 85% (95% CI, 70–93). The 5-year CIF estimates were 31% (95% CI, 30–31%) and 14% (95% CI, 7–29%). The survival curves are shown in Fig. 2.

**Fig. 2:**
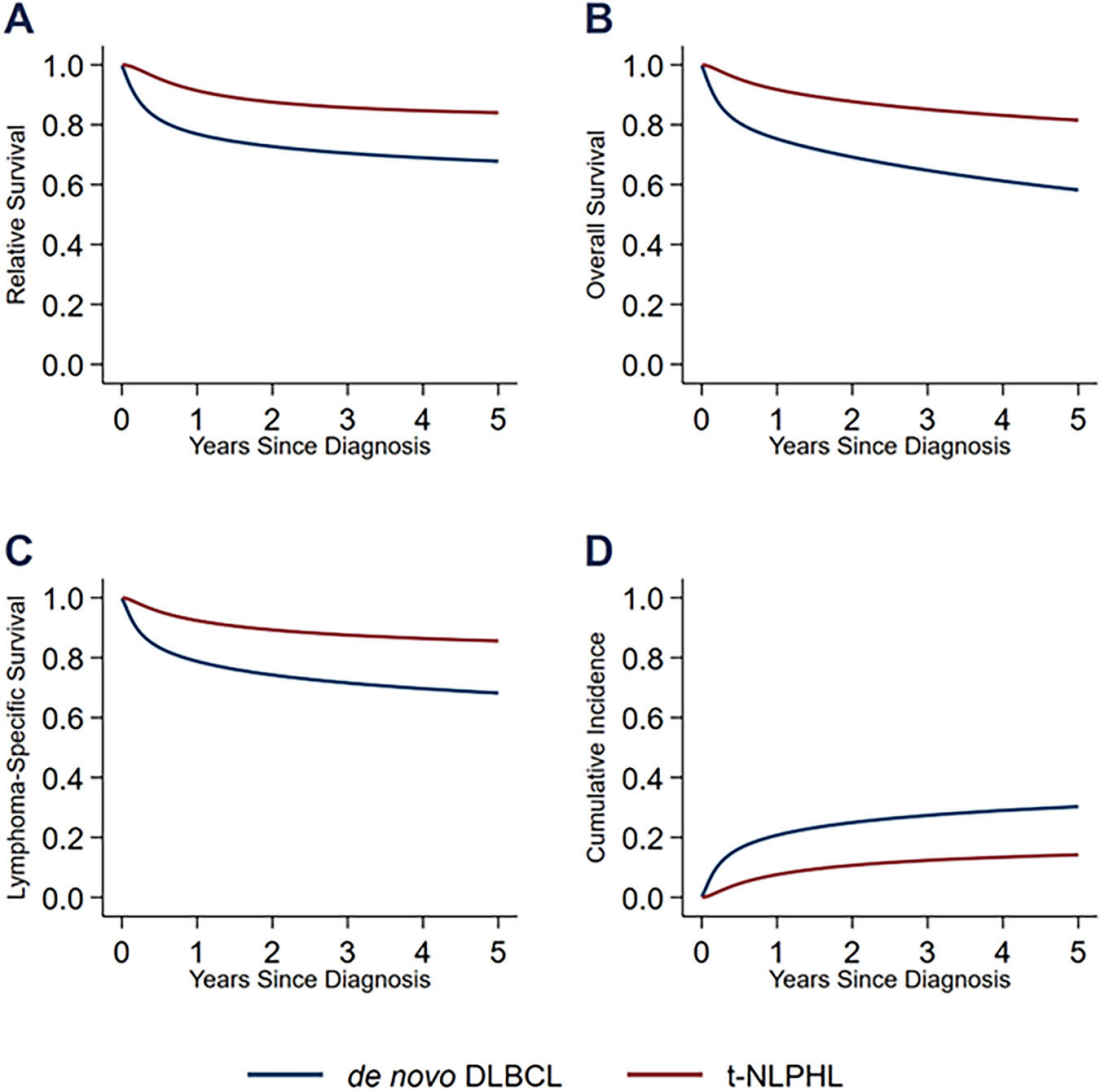
Outcomes of patients with t-NLPHL compared to de novo DLBCL. **A** Relative survival. **B** Overall survival. **C** Lymphoma-specific survival. **D** Cumulative incidence of death from lymphoma.

In order to further compare the survival of t-NLPHL versus de novo DLBCL, we constructed unadjusted and adjusted flexible parametric survival models using HT as the primary exposure and de novo DLBCL as the reference category (Table S2). For RS, the unadjusted and adjusted hazard ratios were 0.28 (95% CI, 0.10–0.82) and 0.29 (95% CI, 0.10–0.88; *P* = 0.03), respectively. For OS, the hazard ratios were 0.26 (95% CI, 0.10–0.66) and 0.33 (95% CI, 0.13–0.87; *P* = 0.03). For LSS, the hazard ratios were 0.30 (95% CI, 0.11–0.85) and 0.37 (95% CI, 0.13–1.04; *P* = 0.06). For the lymphoma-specific CIF, the subdistribution hazard ratios were 0.37 (95% CI, 0.16–0.86) and 0.44 (95% CI, 0.19–1.03; *P* = 0.06). Additionally, we performed an exploratory analysis to assess the impact of prior chemotherapy or radiation therapy on the outcomes of patients with t-NLPHL. There was no significant difference in outcomes between groups (Tables S3, S4).

In this population-level analysis of patients with t-NLPHL in the US, we highlight three key findings. First, the risk of transformation among patients with NLPHL is relatively low compared with other indolent lymphomas. Second, survival significantly worsens following transformation, with patients with t-NLPHL experiencing poorer outcomes than those with non-transformed NLPHL. Finally, unlike patterns observed in other indolent lymphomas, the survival of patients with t-NLPHL is comparable to that of patients with de novo DLBCL.

In our analysis of 44 transformed cases, we observed a cumulative incidence of 2.59% over a median follow-up of 5.58 years. When compared with reported transformation rates for other indolent B-cell lymphomas over similar time frames, NLPHL appears to carry a comparatively lower risk of transformation. For instance, follicular lymphoma (FL) has a reported 5-year transformation rate of 2.8% [10]. Similarly, reported 5-year transformation rates of marginal zone lymphoma (MZL) are higher, with a meta-analysis reporting pooled 5-year transformation rates of 3% for extranodal MZL, 7% for splenic MZL, and 9% for nodal MZL [11], though population-based studies report lower rates, with cumulative incidence ranging from 1.2% at 5 years to 1.7% overall for extranodal MZL and 5.8–14% at 10 years for splenic MZL [2, 12]. Notably, we observed a lower transformation rate for NLPHL than previously reported [4, 6, 7, 9], although this should be interpreted with caution, as apart from one study [8], the median follow-up in our cohort was shorter than in all prior analyses.

Despite the low risk, HT is still a marker of disease progression that confers worse survival. All survival measures were lower compared to non-transformed NLPHL, with 5-year RS decreasing from 97% to 88%. This 9% absolute decrease is consistent with data from large institutional cohorts, showing transformation was associated with poorer prognosis [4, 5, 8]. The biological basis for worse outcomes possibly reflects the acquisition of more complex clonal trajectories [13], thus impacting treatment efficacy.

Most notably, t-NLPHL demonstrated significantly superior outcomes compared to de novo DLBCL, a finding that distinguishes it from transformation patterns observed in other indolent lymphomas. This survival difference persisted after adjustment for demographic and clinical factors, showing approximately 70% lower hazard rates for both RS and OS. These findings contrast with other indolent B-cell lymphomas, where transformation consistently results in outcomes worse than de novo DLBCL. The biological mechanisms underlying this observation are undefined, though t-NLPHL exhibits a distinct molecular profile characterized by the absence of MYC expression, strong host immune response, and upregulation of PD1 [14, 15].

This study has several limitations. First, the sample size was small, with only 44 patients with t-NLPHL included. Second, the SEER database does not capture detailed information on specific treatments, prognostic scores, or molecular and histologic subtypes for either NLPHL or DLBCL. In addition, the exact month and day of diagnosis were unavailable, limiting our ability to accurately determine time to transformation. Finally, the duration of follow-up was shorter than in prior studies, which may have led to underestimation of the true incidence of HT. Nevertheless, this analysis provides essential evidence to establish a contemporary benchmark for transformation risk and outcomes in the modern treatment era.

To our knowledge, this is the first population-level analysis comparing outcomes of t-NLPHL to de novo DLBCL. Our findings show that at the population level, NLPHL has a lower risk of transformation than previously reported in observational studies. Importantly, our analysis confirms that patients who do transform have significantly better survival than those with de novo DLBCL, a novel finding at the population level that builds upon a prior small case series. Larger multi-institutional studies incorporating detailed clinical, pathologic, and molecular data are needed to identify risk factors for transformation, elucidate the biological mechanisms underlying the favorable outcomes observed in t-NLPHL, and determine whether specific treatment strategies can further optimize survival in this unique patient population.

## Supplementary information

Supplementary Appendix

## Data Availability

This study used publicly available data, which can be accessed through the Surveillance, Epidemiology, and End Results (SEER)-17 database.
