# Supplementary material for: Evaluation of incidence and outcomes of transformed nodular lymphocyte predominant Hodgkin Lymphoma in the United States: a population-based cohort study
Source: Blood Cancer J. 2026 Apr 23;16(1):62. doi: 10.1038/s41408-026-01505-y (PMC13106755; doi:10.1038/s41408-026-01505-y)
Supplement: Supplementary file 1 — Supplementary Appendix [file 41408_2026_1505_MOESM1_ESM.docx]

**SUPPLEMENTAL APPENDIX**

**Table of Contents** 1

**Table S1: Patient Characteristics**  2

**Table S2: Univariable and multivariable flexible parametric survival models** 4

**Table S3: Outcomes of tNLPHL by prior receipt of chemotherapy** 5

**Table S4: Outcomes of tNLPHL by prior receipt of radiotherapy**  6

**Table S1. Patient Characteristics**

|  | N=1,700 (%) |
| --- | --- |
| Median age at diagnosis, y (IQR) | 46 (32-59) |
| Age group |  |
| 18-44 | 802 (47%) |
| 45-54 | 323 (19%) |
| 55-64 | 311 (18%) |
| 65-74 | 199 (12%) |
| 75-89 | 65 (4%) |
| Year of diagnosis |  |
| 2010-2012 | 303 (18%) |
| 2013-2015 | 385 (23%) |
| 2016-2018 | 446 (26%) |
| 2019-2022 | 566 (33%) |
| Sex |  |
| Female | 582 (34%) |
| Male | 1,118 (66%) |
| Race |  |
| White | 1,132 (67%) |
| Black | 434 (26%) |
| Other | 108 (6%) |
| Missing | 26 (2%) |
| Ann Arbor stage |  |
| Stage I-II | 1,069 (63%) |
| Stage III-IV | 549 (32%) |
| Missing | 82 (5%) |
| B symptoms |  |
| No B symptoms | 1,197 (70%) |
| B symptoms | 286 (17%) |
| Missing | 217 (13%) |
| Chemotherapy |  |
| No/Unknown | 847 (50%) |
| Yes | 853 (50%) |
| Radiation |  |
| No/Unknown | 1,051 (62%) |
| Yes | 649 (38%) |
| Median time from diagnosis to treatment, d (IQR) | 36 (0-60) |
| Histologic transformation |  |
| No | 1,656 (97%) |
| Yes | 44 (3%) |

Abbreviations: IQR = interquartile range

**Table S2. Univariable and multivariable flexible parametric survival models**

|  | ***de novo* DLBCL** | **t-NLPHL** |  |
| --- | --- | --- | --- |
| RS | HR (95% CI) | HR (95% CI) | P-value |
| Unadjusted | Reference | 0.28 (0.10-0.82) | 0.020 |
| Adjusted | Reference | 0.29 (0.10-0.88) | 0.028 |
| OS |  |  |  |
| Unadjusted | Reference | 0.26 (0.10-0.66) | 0.005 |
| Adjusted | Reference | 0.33 (0.13-0.87) | 0.025 |
| LSS |  |  |  |
| Unadjusted | Reference | 0.30 (0.11-0.85) | 0.023 |
| Adjusted | Reference | 0.37 (0.13-1.04) | 0.060 |
| CIF |  |  |  |
| Unadjusted | Reference | 0.37 (0.16-0.86) | 0.022 |
| Adjusted | Reference | 0.44 (0.19-1.03) | 0.058 |

Abbreviations: CI = confidence interval, CIF = lymphoma-specific cumulative incidence function, HR = hazard ratio, LSS = lymphoma-specific survival, OS = overall survival, RS = relative survival, t-NLPHL = transformed nodular lymphocyte predominant Hodgkin lymphoma

**Table S3. Outcomes of tNLPHL by prior receipt of chemotherapy**

|  | **No Prior Chemotherapy (n=17)** | **Prior Chemotherapy (n=27)** |
| --- | --- | --- |
| 5-year RS (95% CI) | 0.81 (0.48-0.94) | 0.86 (0.65-0.95) |
| 5-year OS (95% CI) | 0.79 (0.49-0.93) | 0.82 (0.63-0.92) |
| 5-year LSS (95% CI) | 0.80 (0.50-0.93) | 0.89 (0.70-0.96) |
| 5-year CIF (95% CI) | 0.20 (0.07-0.50) | 0.11 (0.04-0.30) |

**Table S4. Outcomes of tNLPHL by prior receipt of radiotherapy**

|  | **No Prior Radiotherapy (n=34)** | **Prior Radiotherapy (n=10)** |
| --- | --- | --- |
| 5-year RS (95% CI) | 0.85 (0.65-0.94) | 0.83 (0.43-0.96) |
| 5-year OS (95% CI) | 0.81 (0.62-0.91) | 0.82 (0.46-0.95) |
| 5-year LSS (95% CI) | 0.87 (0.69-0.95) | 0.82 (0.46-0.95) |
| 5-year CIF (95% CI) | 0.13 (0.05-0.30) | 0.18 (0.05-0.54) |
